# Immunomodulatory effects of microbiota-derived metabolites at the crossroad of neurodegenerative diseases and viral infection: network-based bioinformatics insights

**DOI:** 10.3389/fimmu.2022.843128

**Published:** 2022-07-19

**Authors:** Anna Onisiforou, George M. Spyrou

**Affiliations:** Bioinformatics Department, Cyprus Institute of Neurology & Genetics, Nicosia, Cyprus

**Keywords:** microbiota, neurodegenerative diseases, immune system, viruses, microbiota-virus-disease interactions

## Abstract

Bidirectional cross-talk between commensal microbiota and the immune system is essential for the regulation of immune responses and the formation of immunological memory. Perturbations of microbiome-immune system interactions can lead to dysregulated immune responses against invading pathogens and/or to the loss of self-tolerance, leading to systemic inflammation and genesis of several immune-mediated pathologies, including neurodegeneration. In this paper, we first investigated the contribution of the immunomodulatory effects of microbiota (bacteria and fungi) in shaping immune responses and influencing the formation of immunological memory cells using a network-based bioinformatics approach. In addition, we investigated the possible role of microbiota-host-immune system interactions and of microbiota-virus interactions in a group of neurodegenerative diseases (NDs): Amyotrophic Lateral Sclerosis (ALS), Multiple Sclerosis (MS), Parkinson’s disease (PD) and Alzheimer’s disease (AD). Our analysis highlighted various aspects of the innate and adaptive immune response systems that can be modulated by microbiota, including the activation and maturation of microglia which are implicated in the development of NDs. It also led to the identification of specific microbiota components which might be able to influence immune system processes (ISPs) involved in the pathogenesis of NDs. In addition, it indicated that the impact of microbiota-derived metabolites in influencing disease-associated ISPs, is higher in MS disease, than in AD, PD and ALS suggesting a more important role of microbiota mediated-immune effects in MS.

## 1 Introduction

Gut microbiota play an essential role in maintaining homeostasis in the human host, with more than 100 trillion microorganisms (including bacteria, viruses, fungi and archaea) colonizing our gastrointestinal (GI) tract (1, 2). These microbes do not just habitat our GI tract but rather help to regulate various host physiological functions, including metabolic, endocrine and immune functions (3). Gut microbiota have key role in human metabolism as they encode enzymes that are not found in the human genome which are important for the degradation of exogenous and endogenous substrates, resulting in the production of a range of metabolic products (4–6).These microbiota-derived metabolites are key orchestrators of the bidirectional cross-talk that exists between host and microbiota. This molecular dialogue is essential for the regulation of immune responses and the formation of immunological memory, which are important for maintaining body homeostasis and human health.

Symbiosis between commensal microbiota and the immune system is a fine-tuned dynamic process. On the one hand, the immune system plays an important role in maintaining health homeostasis by reacting and eliciting innate immune responses against invading pathogenic organisms such as viral infections, and at the same time maintaining tolerance to beneficial microbiota (7, 8). On the other hand, commensal gut microbiota can regulate and shape immune responses such as influencing various steps of the hematopoiesis process, influence the intestine immune cell population, affecting myeloid cells and lymphoid cell functions and differentiation, thus affecting responses to viral infections (9). In addition, commensal microbiota can also influence the formation of both innate and adaptive memory *via* the production of microbiota-derived bioactive molecules, such as short-chain fatty acids (SCFAs) and neurotransmitters (NTs) (10, 11). However, although several mechanisms have been identified on how commensal microbiota can influence the immune system, the exact mechanisms on how they can affect the formation of innate and adaptive immune memories is still an emerging area of investigation (10).

Perturbations of the microbiome-immune system cross-talk due to microbiome-host dysbiosis can lead to the dysregulation of immune responses against invading pathogens and/or the loss of self-tolerance, leading to systemic inflammation and the genesis of several immune-mediated diseases. Perturbations of microbiome-host symbiosis, resulting in dysbiosis, can be caused by both environmental and genetic factors. However it is believed that the environmental-mediated perturbations outweigh host genetic polymorphism-mediated perturbations in shaping microbiome-host interactions (12).Important environmental factors that can contribute to microbiome-host dysbiosis is diet, antibiotic use, lifestyle (stress) and infection with pathogenic organisms, such as viral infections (13, 14).

Gut microbiota dysbiosis has been associated with the development and/or progression of several NDs, including ALS, AD, MS and PD *via* perturbations of the microbiota-brain-gut axis (15). Microbiota are involved in the production of several metabolic products, such as NTs and SCFAs, which are important for gut-brain communication, brain homeostasis, neurogenesis and neuroinflammation (3, 16–21). Several pathological mechanisms have been suggested by which microbiota can contribute to the development and/or progression of NDs, for example they can increase blood brain barrier (BBB) permeability, augment neuroinflammation and affect the production of several NTs produced by gut microbiota, such as dopamine which is dysregulated in PD (17, 18). However, although gut microbiota dysbiosis is associated with the development of NDs, it remains unknown how ND-associated microbiota profiles contribute to neuroinflammation and lead to the development of specific disease phenotypes.

Interestingly, evidences also suggests that commensal microbiota can suppress or promote certain viral infections *via* microbiota- mediated direct or indirect mechanisms (13, 22). The development of several NDs is associated with numerous viral infections (23–25), hence this bidirectional interaction between viruses and commensal microbiota could possibly contribute to the onset or progression of a ND. According to the “multiple hit” hypothesis the development of NDs requires the combinatorial action of multiple ND-associated risk factors (26). However, it still remains undetermined how the combinatorial effect of multiple risk factors contributes to disease development. Therefore, as both microbiota and viral infections are environmental factors associated with NDs, it is important to investigate how microbiota-virus interactions might contribute to ND development.

Network-based approaches have been extensively utilized to provide insight into microbe-host interactions, through the analysis of various single-omics data including proteomics, transcriptomics and metabolomics (27–30).Various network-based methodologies have been employed to analyze microbe-host interaction networks, with the most common methods being topological analysis, such as modules and community detection, as well as functional analysis (31, 32). In addition, some studies have used multi-omics approaches to investigate pathogen-host interactions (33). However, only a few studies used multi-omics network approaches to investigate microbiome data (27). Moreover, several studies have used network approaches to investigate microbe-microbe interactions, with the majority investigating bacteria-to-bacteria interactions, whereas only a few studies have investigated viral or fungal interactions (27, 34). Although several studies have used network approaches to investigate the role of viruses in the triggering of diseases, there is a lack of studies that focus specifically in the development of NDs (35). In our previous work, we have developed our own integrative network-based bioinformatics methodology which was utilized to identify viral-mediated pathogenic mechanisms by which viruses associated as risk factors for MS could lead to its development, through virus-host protein-protein interactions (PPIs) (36).

In this paper, we extend upon the methodology developed in our previous work (36) and use an integrative multi-omics network-based bioinformatics approach to investigate the immunomodulatory effects of both microbiota (bacteria and fungi) and viruses in general and ND specific states. To our knowledge, this is the first study that uses network-based approaches to investigate how microbiota-virus interactions might contribute to NDs development. We first, investigate the contribution of the immunomodulatory effects of microbiota (bacteria and fungi) in shaping immune responses and influencing the formation of immunological memory cells *via* the production of their metabolic products. We aim to also identify microbiota components which, through their immunomodulatory effects, might be able to influence ISPs involved in the pathogenesis of NDs. In addition, as a case study, we explore how microbiota-Epstein-Barr Virus (EBV)-immune system interactions might facilitate the development and/progression of several NDs.

## 2 Methods

**Figure 1:**
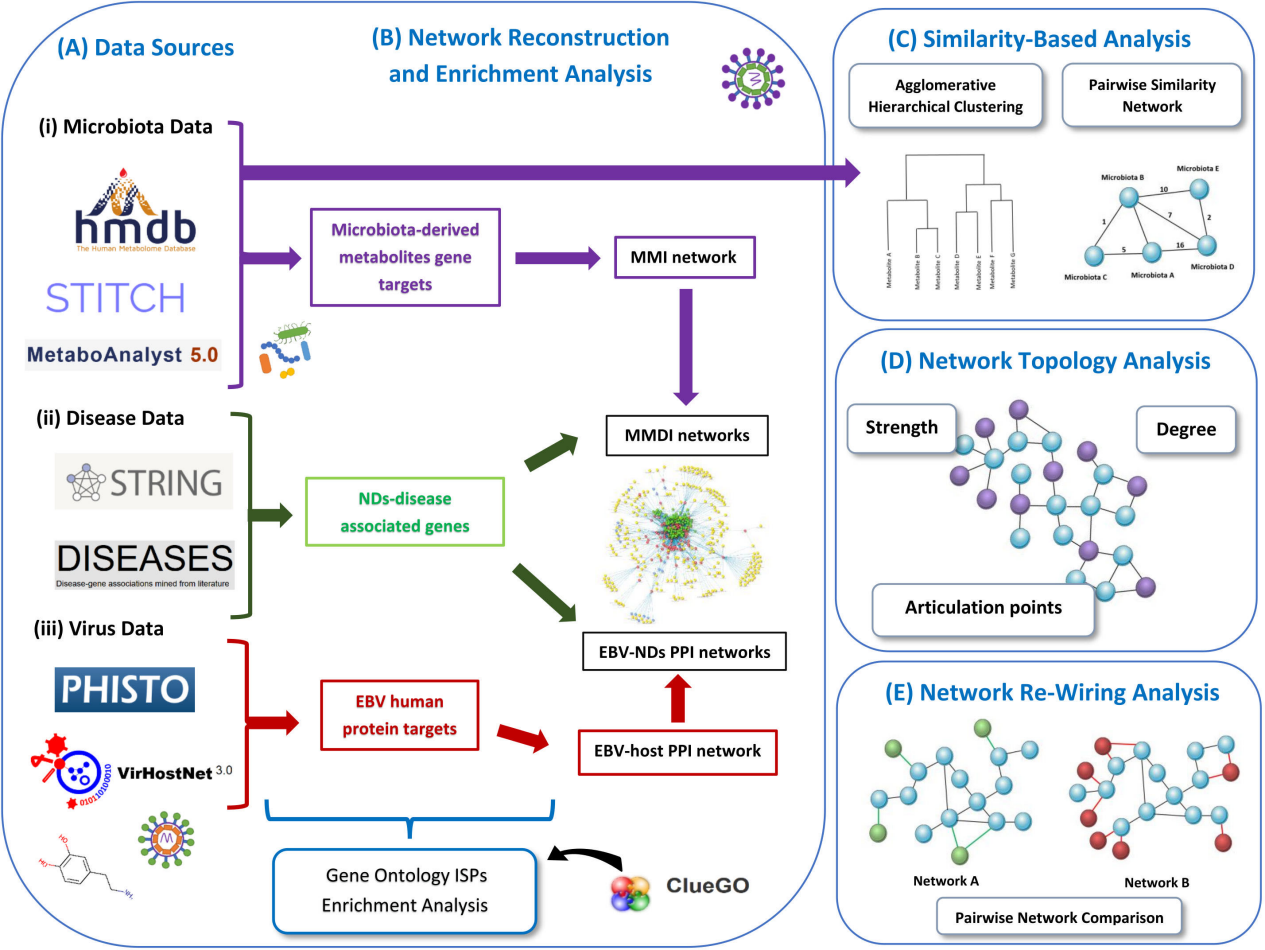
Schematic representation of our methodology used in this paper. We present the various data sources that we used to collect the data **(A)**. Then we represent the data used for enrichment analysis and how the networks are reconstructed, including the MMI network, MMDI networks, EBV-ND PPI networks and EBV-host PPI network **(B)**. Then we also present the two similarity-based analysis methods used to analyze the data, the agglomerative hierarchical clustering analysis and the pairwise similarity network analysis **(C)**. We also indicate the three network topology metrics (degree, strength and articulation points) **(D)**, that were used to analyze the pairwise similarity network (microbiota-to-microbiota association network). Finally, we present the re-wiring analysis that was used to perform pairwise network comparison between the MMDI networks **(E)**.

Our pipeline approach consists of the following steps: (A) Collection from well-known Data Sources, (B) Network Reconstruction and Enrichment Analysis, (C) Similarity-Based Analysis, (D)Network Topology Analysis, and (F) Network Re-Wiring Analysis. **Figure 1** illustrates the main components of our methodology.

### 2.1 Data sources

#### (i) Collection of microbiota data

To investigate the effects of microbiota-derived metabolic products on ISPs we first mined the Human Metabolome Database (HMDB) (version 4.0) (37) to collect microbiota derived-metabolites found in the feces, blood and cerebrospinal fluid (CSF) of human samples. We used an in-house developed R script to parse the feces, serum and CSF xml datasets (datasets downloaded in xml format) from the HMDB with the aim to identify and extract only the metabolites that had biological disposition in bacteria and/or fungi (release date:2020-09-10). Specifically, for each of the metabolite’s data contained within each of the three xml datasets, our script examined whether the metabolite in their biological disposition section was containing the terms “Fungi” or “Bacteria”. If these terms were present in the metabolite data, the algorithm classified the corresponding metabolites as associated to fungi and/or bacteria and it also extracted the names of these fungi or bacteria, if they were available Overall, from the feces, serum and CSF xml datasets, after removing duplicates, we identified 188 metabolites that were indicated to be produced by bacteria and fungi. From these 188 metabolites, 157 were indicated to be produced only by bacteria and 11 to be produced only by fungi, whereas 20 of them were indicated to be produced by both bacteria and fungi. Based on the data extracted after parsing the xml datasets from HMDB, we were able to collect the names of microbiota that produce most of the 188 identified microbiota-derived metabolites, which included either species or strains or genera names. More specifically, we extracted 397 names (genera/species/strains) of bacteria or fungi which were associated with 138 out of the 188 identified microbiota-derived metabolites. However, although the other 50 of the microbiota-derived metabolites were indicated to have biological disposition in bacteria or fungi, no specific names were provided by the HMDB database. From these 50 microbiota-derived metabolites, 49 were indicated to be produced by bacteria and 1 by fungi. Therefore, in total we extracted 671 microbiota names-to-metabolite associations, including the 50 microbiota-derived metabolites without associated names, which were indicated under the general annotation of bacteria or fungi associated.

Through the above-mentioned parsing of HMDB, we generated metabolite-to-gene associations by collecting the associated genes for each metabolite. For the same set of the 188 metabolites, we also collected metabolite-to-gene associations using the *MetaboAnalyst* tool (version 5.0) (38), that provides metabolite-to-gene associations which are extracted from STITCH (‘search tool for interactions of chemicals’) database (39). Then we combined the metabolite-to-gene associations obtained from HMDB and STITCH databases, and we removed duplicate entries. These resulted in 5931 metabolite-to-gene associations between 130 out of the 188 microbiota-derived metabolites and 2085 human genes, whereas no metabolite-to-gene association data were available for the rest 58 microbiota-derived metabolites.

#### (ii) Collection of NDs disease-associated data

Using the same approach as in our previous work (36), we obtained disease-associated gene data for each of the four NDs (ALS, AD, PD and MS) using the S*TRING: disease* query of the StringApp in Cytoscape, which is a network visualization and analysis tool (40). The *STRING: disease* app allows to easily import disease-associated data which are extracted from the DISEASES database (41) into Cytoscape, which automatically creates a PPI disease network. We obtained the top 200 highly disease-associated genes, which had the highest disease association score, for each of the four NDs. The confidence cut-off score of interactions between the human proteins was set at 0.8 for all four ND disease-associated networks. The confidence cut-off score determines the quality of the evidence on whether the interaction between the proteins is likely to be true, with confidence values ranging from 0 (low) to 1.0 (high) (42).

#### (iii) Collection of viruses-host PPI data

Virus-host PPI data for EBV were obtained from PHISTO (43) and VirHostNet 3.0 (44) databases, which provide experimentally validated pathogen-host PPIs. Then we combined the virus-host PPIs data that we obtained from these two databases and removed duplicated entries, resulting into 7069 EBV-human host PPIs. The data contains 153 EBV proteins from four EBV viral strains (GD1, B95-8, AG876, HHV4 type 2) that target 1247 human proteins. Based on its genome, EBV has been found to have a coding potential of around 80 viral proteins, however not all proteins have yet been identified. The generated EBV-human host PPIs dataset contains 48, 59, 45 and 1 viral proteins from the EBV strains GD1 (taxid id:10376), B95-8 (taxid id: 10377), AG876 (taxid id: 82830) and HHV-4 type 2 (taxid id: 12509), respectively.

### 2.2 Network reconstruction and enrichment analysis

For the purpose of this investigation, using the data collected above we reconstructed and visualized the following three types of networks:

#### (i) Microbiota - metabolites - GO ISP terms network

To model the immunomodulatory effects of microbiota-derived metabolites produced by bacteria and fungi, we constructed a microbiota - metabolites -Gene Ontology Immune System Processes (GO ISPs) network, called MMI network, using *visNetwork* package in R (45). The constructed network contains three types of nodes, (a) microbiota (bacteria and fungi), (b) metabolites and (c) GO ISPs terms. It also contains three types of edge interactions between the nodes: (a) microbiota-to-metabolite, (b) metabolite-to-GO ISP and (c) GO ISPs-to-GO ISPs. To identify the GO ISPs that are modulated by microbiota-derived metabolites, we performed enrichment analysis on the 2085 human genes that are targeted by the 130 microbiota-derived metabolites. Enrichment analysis was performed using the ClueGO app (46) in Cytoscape using the GO ISPs database and keeping only significantly enriched terms with adjusted *P* -value ≤0.05 (corrected with Benjamini-Hochberg). Then the enriched GO ISPs terms were merged with the microbiota-to-metabolite associations obtained from the HMDB database, in order to construct the MMI network. **Table 1** indicates the characteristics of the MMI network, which is composed of 472 nodes and 3770 edges.

**Table 1:** Characteristics of the MMI network.

	Number of nodes/edges
**Nodes**	
Microbiota nodes	259
Metabolite nodes	93
GO ISPs nodes	120
**Total**	**472**
**Edges**	
Microbiota-to-Metabolite	437
Metabolite-to-GO ISPs	3005
GO ISPs-to-GO ISPs	328
**Total**	**3770**

#### (ii) Microbiota-metabolites-ND associated genes- GO ISPs networks

To identify disease-associated genes that can be modulated by microbiota components in each of the four NDs (ALS, MS, AD and PD), we merged the microbiota-metabolites-genes interactions, collected in Section 2.1 (i), with each of the 200 disease-associated genes of these diseases. For each of the four NDs we then constructed a microbiota-metabolites-ND associated genes- GO ISPs (MMDI) network. More specifically, to create the four MMDI networks we first performed enrichment analysis on the 200 disease-associated genes for each of the four NDs, using the same parameters as in the enrichment analysis performed in part (i). Then we merged the microbiota-metabolites-genes interactions with the ND-associated genes and the ND siginificantly enriched GO ISP terms, allowing to construct the four MMDI networks. Then for each of the four MMDI networks we only retained microbiota components and metabolites that interact with the ND-associated genes. This allowed to identify microbiota components which produce metabolites that target genes associated with these diseases and therefore can modulate ND-associated GO ISPs. The characteristics of each of the four MMDI networks are described in **Table 2**.

**Table 2:** Characteristics of the MMDI networks of MS, PD, ALS and AD.

	Number of nodes/edges			
**Nodes**	**MS**	**PD**	**ALS**	**AD**
Microbiota nodes	94	108	84	104
Metabolite nodes	27	30	26	29
Gene nodes	50	30	22	32
GO ISP nodes	295	63	28	95
**Total**	**466**	**231**	**160**	**260**
**Edges**				
Microbiota-to-Metabolites	119	147	107	133
Metabolites-to-Genes	179	136	97	141
Genes-to-GO ISPs	1680	283	117	515
Genes-to-Genes	313	88	83	148
GO ISPs-to-GO ISPs	3104	251	41	609
**Total**	**5395**	**905**	**445**	**1546**

#### (iii) EBV-ND PPI Networks

To investigate the possible contribution of microbiota - virus interactions in the development of NDs, we used the case of EBV, which has been associated with the development of three out of the four selected NDs, namely AD, MS and PD (47–49). The development of ALS was not strongly associated with EBV and hence was excluded from this part of the analysis. First, we created an EBV-host PPI network in Cytoscape using the virus-host PPI data collected in Section 2.1 (iii), resulting in a network of 1400 nodes and 7069 edges. We then performed enrichment analysis on the 1247 human protein targets of EBV proteins in the EBV-host PPI network, using the same parameters as before, with the aim to identify GO ISPs that can be modulated by EBV.

In order to identify the immunomodulatory effects of EBV in the three remaining NDs, we merged the EBV-host PPI network with each of the 200 disease-associated proteins (from the corresponding genes), resulting in the construction of three integrated EBV-ND PPI networks. Then we performed enrichment analysis on the human protein targets of EBV and their first neighbors, extracted from each of the three integrated EBV-ND PPI networks. The human proteins used to perform enrichment analysis were 1392, 1389 and 1381 for AD, PD and MS respectively.

### 2.3 Similarity-based analysis

#### (i) Microbiota and microbiota-derived metabolites based on GO ISPs interactions

**Figure 2:**
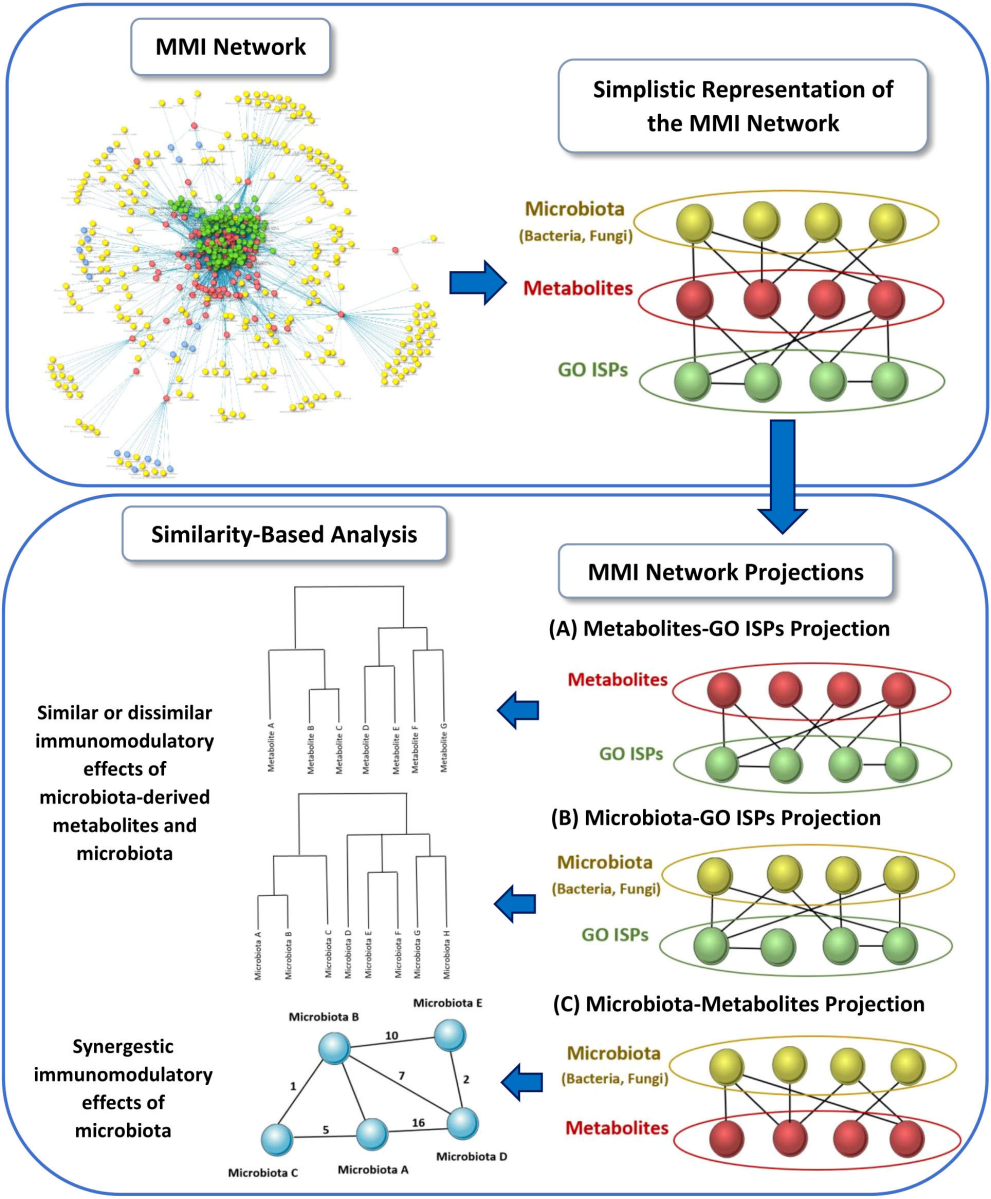
Schematic representation of the three network projections extracted from the MMI network, that involves the direct relationships between: **(A)** metabolites and GO ISPs, **(B)** microbiota and GO ISPs and **(C)** microbiota and metabolites. For each projection the illustration indicates the main methodology used and the output obtained from each projection.

Using the MMI network, we extracted three network projections that involve the direct relationships between (A) metabolites and GO ISPs, (B) microbiota and GO ISPs and (C) microbiota and metabolites, illustrated in **Figure 2**. From the MMI network we isolated the edge interactions between metabolites and GO ISPs, and the edge interactions between microbiota and metabolites, to generate the two projections respectivelly, namely the (A) metabolites and GO ISPs and the (C) microbiota and metabolites. In addition, in order to extract the projection (B) microbiota-to-GO ISPs, we had to first identify for each microbiota in the MMI network all the connected metabolites and also identify the GO ISPs that interact with these metabolites. For the last two network projections, (B) and (C), we only retained microbiota with known genera/species/strain name, thus removing microbiota with unknown name. Then, we performed similarity-based analysis to identify clusters of (A)microbiota-derived metabolites, shown in **Figure 2A**, and (B) microbiotas (genera/species/strains), shown in **Figure 2B**, that have similar or dissimilar immunomodulatory effects, based on the GO ISPs that they can modulate. By using the two projections that involve the direct relationships between (A) metabolites and GO ISPs and (B) microbiota and GO ISPs we created two binary matrices. The microbiota to GO ISPs matrix included 256 microbiota and 120 GO ISPs, whereas the microbiota-derived metabolites to GO ISPs included 73 metabolites and 120 GO ISPs. Then by using the *vegan* package in R (50) we measured the similarity between metabolites and microbiota based on GO ISP terms effects using the Jaccard similarity index. Then, by using the *factoextra* package in R (51) we performed agglomerative hierarchical clustering on the two projection similarity results, using the average method. Agglomerative hierarchical clustering is an unsupervised clustering method that allows to group objects into clusters based on their similarity. It works very well for small datasets and allows to easily extract information from the analyzed data. There are several methods that can be used to compute the distance between the clusters in hierarchical clustering, such as “average”, “single” or “median” methods. In analyzing these datasets, we used the “average” method because it had the highest correlation coefficient value between the cophenetic distance (distance in the vertical axis of the tree) and the original distance metric used for the clustering. This was a marker of validity of the clustering method that we used. The similarity-based analysis that was performed on the metabolites and GO ISPs projection, **Figure 2A**, and the microbiota to GO ISPs projection, **Figure 2B**, were used to identify metabolites and microbiota, respectively, which had similar or dissimilar immunomodulatory effects.

#### (ii) Synergestic relatioships between microbiota components in modulating ISPs

Microbiota components that produce the same metabolites influence the same ISPs and thus have the same effects on those processes. Thus, we used the direct relationships of microbiota components and their metabolic products, **Figure 2C**, to investigate the possible presence of synergestic actions between microbiota components in modulating ISPs. To identify the microbiota components that can affect the same GO ISPs we created a microbiota-to-microbiota associations network, based on pairwise similarity using List2Net (https://c-big.shinyapps.io/list2net/), a shiny application created by our group, that transforms lists of data to networks based on pairwise commonalities. Therefore, within the network, a microbiota component is connected with an edge with another microbiota component based on the common metabolites they produce. The weight of each edge is the sum of the common metabolites between each two connected microbiota components. The application also provides network metrics of various topological analysis measures, including degree and strength centralities. The degree centrality indicates the number of connections between two microbiota components and it was used to identify the microbiota components that have the highest number of associations with other microbiota components. The strength centrality, indicates the sum of the weights of all the links each node has and it was used to identify the Top 10 microbiota components with the highest potential to exert synergistic ISP effects. In addition, List2Net also provides other network metrics, such as identifying articulation points within the network whose removal will disconnect the largest connected microbial module within the network into several smaller modules. The articulation points metric was used to identify microbiota components within the network whose presence was important for ensuring network connectivity.

#### (iii) EBV and microbiotas common ISP effects in general and ND specific states

We used a Venn diagram, to compare the 53 EBV significantly enriched GO ISP terms obtained from the EBV-host PPI network with the 120 GO ISP terms associated with microbiota from the MMI network. The comparison revealed 24 common GO ISPs that can be modulated by EBV and microbiota components. The number of the microbiota components that co-modulate with EBV these 24 GO ISPs was found to be 241. Similarity-based analysis was performed between the 241 microbiotas and EBV based on their similarity to modulate the 24 common GO ISPs, by using the same methodology as in part (i). The similarity-based analysis was used to arrange the 241 microbiotas based on how similar or dissimilar immunomodulatory effects they had in respect to these 24 common GO ISPs with EBV.

In addition, we compared the EBV significantly enriched GO ISPs per disease (AD, MS and PD) with the GO ISPs that have been identified to be modulated by microbiota components in the corresponding MMDI networks identified in Section 2.2 parts (ii) and (iii). This allowed to identify ND-associated GO ISPs that can be modulated by both microbiota components and EBV.

### 2.4 Network re-wiring of the immunomodulatory effects of microbiota in ND states

Microbiota-host interactions are not static, but rather a dynamic process that changes in different conditions, such as in disease states. Network re-wiring allows to capture how the connectivity of molecular interactions changes under different disease states. Therefore, to investigate the role of microbiota-host interactions in influencing ISPs in the four NDs, we used the *DyNet* app in Cytoscape which allows to visualize and analyze dynamic changes in molecular interactions in multi-states (52, 53). The *DyNet* app performs pairwise network comparison, where two networks are compared based on their similarity of their nodes and edges. Therefore, it allows to identify nodes and edges which are present in both networks and are indicated in white color, **Figure 7**. It also indicates nodes/edges that are only present within one of two networks, indicated in the one network as red and in the other as green, **Figure 7**. Network-rewiring analysis allows to identify differences and commonalities of the interaction of microbiota with the GO ISPs associated with the four ND states.

## 3 Results

### 3.1 Comparison between bacterial-versus fungi-derived metabolites

**Figure 3:**
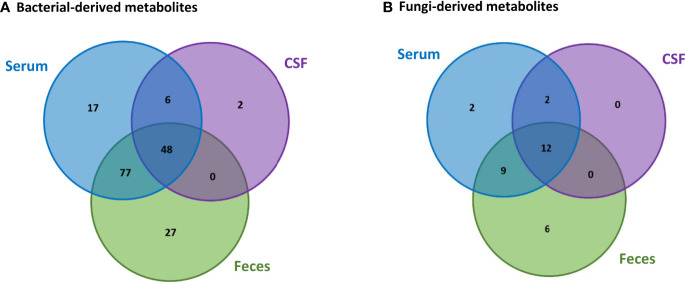
Comparison of the **(A)** bacterial-derived and **(B)** fungi-derived metabolites found in the feces, serum and CSF human samples, obtained from the HMDB database.

Based on the data collected from the HMDB, we were able to identify 188 metabolites in the feces, serum and CSF human samples that can be produced by bacteria and fungi. From these metabolites, 157 can be produced only by bacteria, 11 can be produced only by fungi, whereas 20 can be produced by both bacteria and fungi. Comparison between the bacterial-derived metabolites found in the different human samples, **Figure 3A**, indicates that from the 152 metabolites that were found in the feces samples, 125 can also be found in the serum samples and 48 of these metabolites are also in the CSF samples. This possibly suggests that 125 metabolites from the gut can pass into the bloodstream (systemic circulation) and then 48 of these metabolites can also reach the brain. In addition, comparison between the fungi-derived metabolites found in the different human samples, **Figure 3B**, indicates that 21 of the metabolites found in the gut (feces samples) can enter into the bloodstream and then 12 of these metabolites can also possibly reach the brain.

However, we also observe that some bacterial-derived metabolites, **Figure 3A**, are not found in the feces samples. More specifically, 17 metabolites are only found in the serum, 6 both in the serum and CSF and 2 only in the CSF. Also, similarly few fungi-derived metabolites, **Figure 3B**, are not found in the feces samples. Specifically, 2 metabolites are only found in the serum and 2 metabolites are found in both the serum and CSF. This is probably because these microbiota-derived metabolites are not produced by microbiota located in the gut, but are rather produced by other commensal microbiota in other tissues.

### 3.2 GO ISPs associated with the gene targets of the microbiota-derived metabolites

The GO ISPs enrichment analysis results of the 2085 human genes that are targeted by the 130 microbiota-derived metabolites revealed 120 statistically significant GO ISPs, which are associated with 542 out of the 2085 of the human genes used for enrichment analysis, **Table 3**. These 542 human genes are targeted by 93 microbiota-derived metabolites, of which 81 are known to be produced only by bacteria and 12 are produced by both bacteria and fungi.

**Table 3:** GO ISPs enrichment analysis results of the 2085 human gene targets of the 130 microbiota-derived metabolites indicating the % of terms that belong into each functional group.

Functional groups of GO ISPs terms	Percentage of Terms per group
positive regulation of leukocyte migration	20.49
complement activation, classical pathway	14.75
antigen receptor-mediated signaling pathway	11.48
humoral immune response mediated by circulating immunoglobulin	10.66
myeloid leukocyte differentiation	7.38
neutrophil mediated immunity	6.56
positive regulation of B cell activation	4.92
neutrophil-mediated killing of symbiont cell	3.28
cellular response to interferon-gamma	2.46
negative regulation of inflammatory responses to antigenic stimulus	2.46
negative regulation of cellular extravasation	2.46
hematopoietic stem cell differentiation	2.46
complement receptor mediated signalling pathway	1.64
antimicrobial humoral immune response mediated by antimicrobial peptide	1.64
defense response to Gram-negative bacterium	1.64
microglial cell activation	1.64
eosinophil migration	1.64
antigen processing and presentation of exogenous peptide antigen	0.82
myeloid dendritic cell chemotaxis	0.82
megakaryocyte differentiation	0.82

The GO ISPs results, shown in **Table 3**, indicated that 20.49% of the GO ISP terms belong to the group of positive regulation of leukocyte migration and 7.38% belong to the group of myeloid leukocyte differentiation. This indicates that microbiota can regulate and shape myeloid and lymphoid cells function and differentiation, which is also supported by existing literature (9). For example, microbiota-derived metabolites were shown to be able to stimulate the migration of neutrophiles at the site of inflammation or injury (54–56).

In addition, 14.75% of the enriched terms belong to the group of complement activation, classical pathway that involves processes that lead to the negative or positive regulation of the complement system *via* activation through the classical pathway. The results of our analysis is consistent with existing evidence indicating that pathogenic bacteria and commensal microbiota can hijack regulatory proteins of the complement system (57) and its activation (58). The complement system plays an important role in innate immune system defenses against pathogens and it also complements antibody responses against pathogens by the adaptive immune system (59). It also plays a critical role in commensal microbiota-immune system symbiosis and health homeostasis (60, 61). Improper complement system recognition of commensal microbiota as pathogenic will result in excessive immune responses and lead to the emergence of immune-mediated diseases (60, 61). Therefore, the ability of microbiotas to modulate the classical complement pathway indicates that they can affect both innate and adaptive immune responses against opportunistic pathogens and improper activation of this system can contribute to disease emergence.

The enrichment analysis results also indicated that 11.48% of the enriched GO ISPs terms belong to the group of antigen receptor-mediated signaling pathway, that involves the molecular signals that are initiated by the cross-linking of an antigen receptor on B or T cells. This pathway is essential for the activation of B-cells and their differentiation into either short-lived plasma cells that produce and secrete antibodies or memory B cells (62). In addition, this pathway is also essential for the activation of T cells and their differentiation into effector T cells that have different functions: cytotoxic T cells, helper T cells, regulatory T cells and memory T cells (63). Therefore, the results of our analysis indicate that microbiota have the ability to modulate through metabolite -gene interactions the signaling pathways that are involved in the activation and differentiation of B and T cells and thus the formation of immunological memory.

Moreover, 10.66% of the enriched terms belong to the group of humoral immune response mediated by circulating immunoglobulin, which involves immune responses mediated by antibodies produced by plasma B cells. Interestingly, 1.64% of the terms belong to the group of antimicrobial humoral immune response mediated by antimicrobial peptides, which suggests that microbiota can also specifically modulate antibody immune responses against microbes. In addition, 3.28% of terms belong to the group of neutrophil-mediated killing of symbiont cell that involves the direct killing by a neutrophil of symbiotic microbiota. Therefore, microbiota can upregulate or downregulate humoral immune responses against invading pathogens and also modulate ISPs that are involved in host-symbionts interactions. This is supported by evidence that indicates that gut commensal microbiota can affect antibody production, particularly immunoglobulin A (IgA), and the production of autoantibodies (64, 65).

Furthermore, 1.64% of the terms belong to the group of microglia cell activation, which are considered as the resident macrophages of the CNS and play an important role in inflammation and infection within the brain (66). Therefore, the results of our analysis indicate that microbiota can also affect the regulation of innate immune responses in the CNS. This is supported by experimental evidence indicating that microbiota can affect the maturation and activation of microglia cells through the production of SFCAs and NTs (67, 68).

### 3.3 Similarity-based analysis results on the immunomodulatory effects of microbiota and their metabolites

**Figure 4:**
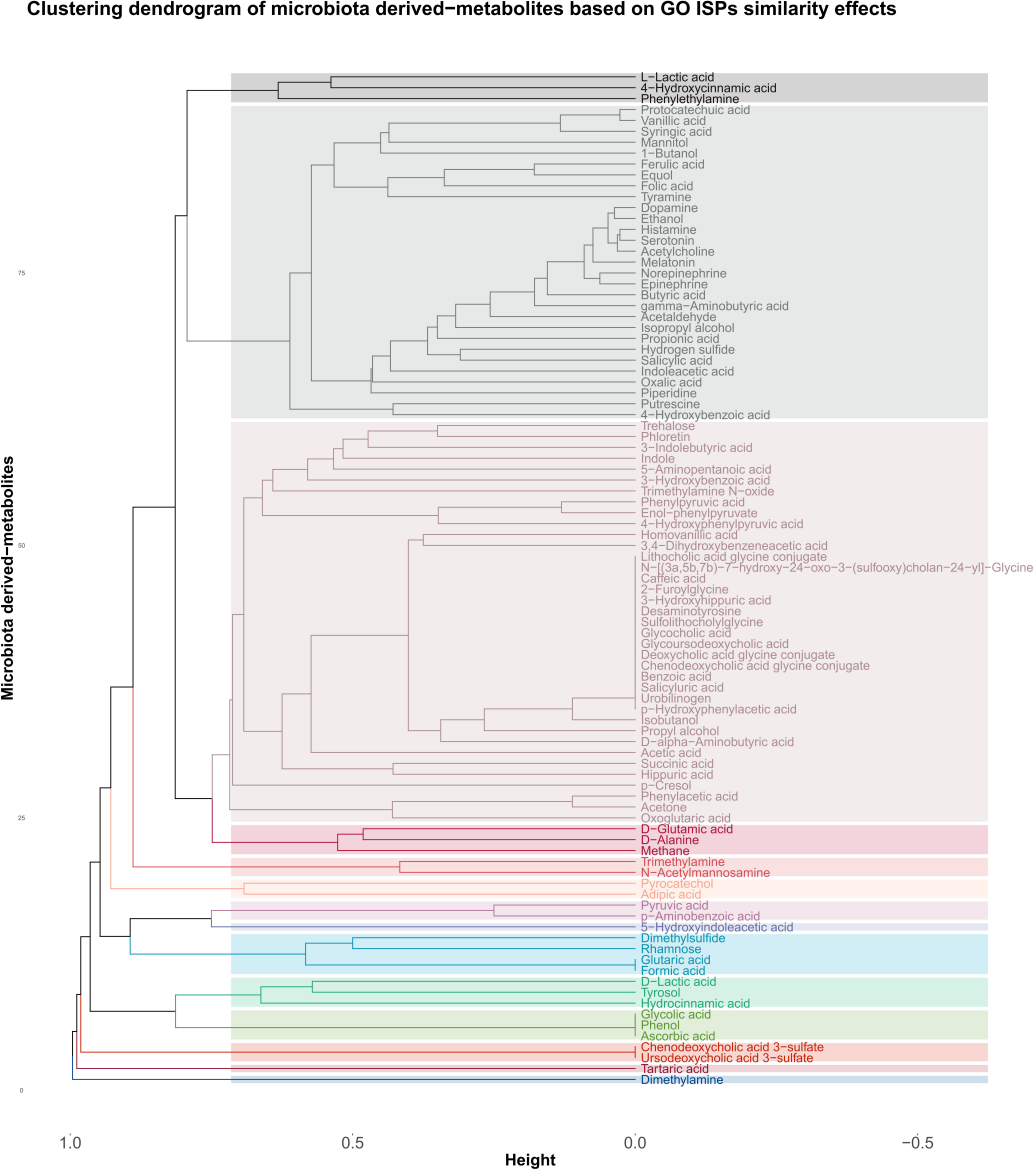
Clustering dendrogram of the 93 metabolites that target the 542 genes that were found to be associated with the 120 GO ISPs, based on their interaction similarity with GO ISPs.

The agglomerative hierarchical clustering of the 93 microbiota-derived metabolites that interact with the 542 genes that are associated with the 120 GO ISPs, indicated the presence of 14 clusters of metabolites based on similarity of GO ISP effects, shown in **Figure 4**. The clustering analysis results showed that several NTs, such as acetylcholine, gamma-aminobutyric acid, serotonin, norepinephrine, epinephrine, histamine and dopamine belong to the same cluster and descent from the same branch indicating that they exert similar immunomodulatory effects. Interestingly, acetaldehyde metabolite which is a byproduct of alcohol and the metabolite ethanol also belong to the same cluster and descent from the same branch as these NTs. Also, the hormone metalonin and the gasotransmitter hydrogen sulfide belong the the same cluster. In addition, the SCFAs butyric acid,proprionic acid, and salicylic acid which is the major metabolite of aspirin are also in the same cluster. This indicates that these microbiota-derived metabolites can exert similar immunomodulatory effects.

Whereas, contrary to formic acid and proprionic acid, acetic acid which is also an SCFA, belongs in a distant cluster, inidicating dissimilar immunomodulatory effects between acetic acid and these two SCFAs. Disimilar effects can also be seen between L-lactic acid and its harmful enantiomer D-lactic acid, where the former is in the same cluster as 4-hydroxcinammic acid and the neurotransmitter phenylethylamine. Whereas, D-lactic acid is in a distant cluster and exert similar effects with the metabolites hydrocinammic acid, which is a carboxylic acid, and tyrosol, which is a phenolic antioxidant compound that can be found in natural sources, such as wine an virgin olive oil.

Moroevoer, the clustering results indicated that several metabolites can exert exactly the same immunomodulatory effects, such as the SCFA formic acid which exerts exactly the same ISP effects as flutaric acid. Exactly the same immunomodulatory effects can also be observed between the metabolites phenol, glycolic acid and ascorbic acid, also known as vitamin C.

Furthermore, the agglomerative hierarchical clustering results of the 256 microbiotas based on GO ISP terms similarity indicated the presence of 14 clusters. The results of the agglomerative hierarchical clustering can be found in **Figure 1** of **Supplementary File 1**. The analysis results indicated that several microbiotas can affect exactly the same ISPs *via* their metabolic products. This suggests that groups of microbiota can influnce the same ISPs, however it does not necessarily mean that their effect on these processes is the same as they might Interact with different genes. Therefore, the composition of the metabolites they produce and their respective gene targets will determine the outcome of the interaction between a microbiota and an ISP, resulting in its activation or inhibition.

### 3.4 Synergistic combinatorial effects of microbiota components on ISPs

To identify modules of microbiota that produce the same metabolites and thus affect the same ISPs, we created a microbiota-to-microbiota associations network (256 nodes, 2654 edges) based on pairwise similarity of the 72 metabolites the 256 microbiotas (genera/species/strain) produce. Visualization of the network indicated that the majority of microbiota components produce at least one metabolite that is also produced by another microbiota component, forming a large connected microbial module. This suggests that several pairwise groups of microbiota components can modulate ISPs *via* similar mechanisms through their metabolic products, resulting in synergistic immune mediated effects. In addition, based on the constructed network, only 6 microbiota components produce unique metabolites. The network is also composed by two small connected microbial modules. The first one contains only methane producing bacteria, known as methanogens, thus these microbiota components modulate the immune system in the same way. The second microbial module contains two *Lactobacillus* species, the *Lactobacillus plantarum* and *Lactobacillus paracasei*, where they share 1 metabolite that is not shared by other microbiota components.

In addition, network analysis of the microbiota-to-microbiota associations network, revealed 9 microbiota components that can act as articulation points within the network, namely: *Clostridium beijerinckii* species, *Pseudomonas putida* species, *Micrococcus* genus, *Klebsiella* genus, *Pseudomonas fluorescens* species, *Escherichia coli* species, *Lactobacillus* genus, *Corynebacterium glutamicum* species and *Alcaligenes* genus. Articulation points are nodes within the network, whose removal will disconnect the largest connected microbial module into several smaller modules, thus these microbiota components are essential for ensuring network connectivity. Some of the identified articulation points are known opportunistic pathogenic organisms, such as *Escherichia coli* species, *Klebsiella* genus, *Alcaligenes* genus and *Pseudomonas putida* species. Whereas, other articulation points, such as *Lactobacillus* genus, are known beneficial gut microbiota, which are also found in probiotic supplements. *Pseudomonas fluorescens* species, which act as an articulation point, reside in low levels in various body sites and are considered non- pathogenic. However, they can cause acute opportunistic infection in rare occasions, and interestingly they have been associated with the development of Chron’s disease (69, 70). In addition, *Clostridium beijerinckii* is also a non-pathogenic clostridia species, unlike other known clostridia species, like C*lostridium difficile* and *Clostridium botulinum* that are known human disease-causing pathogens (71).

**Figure 5:**
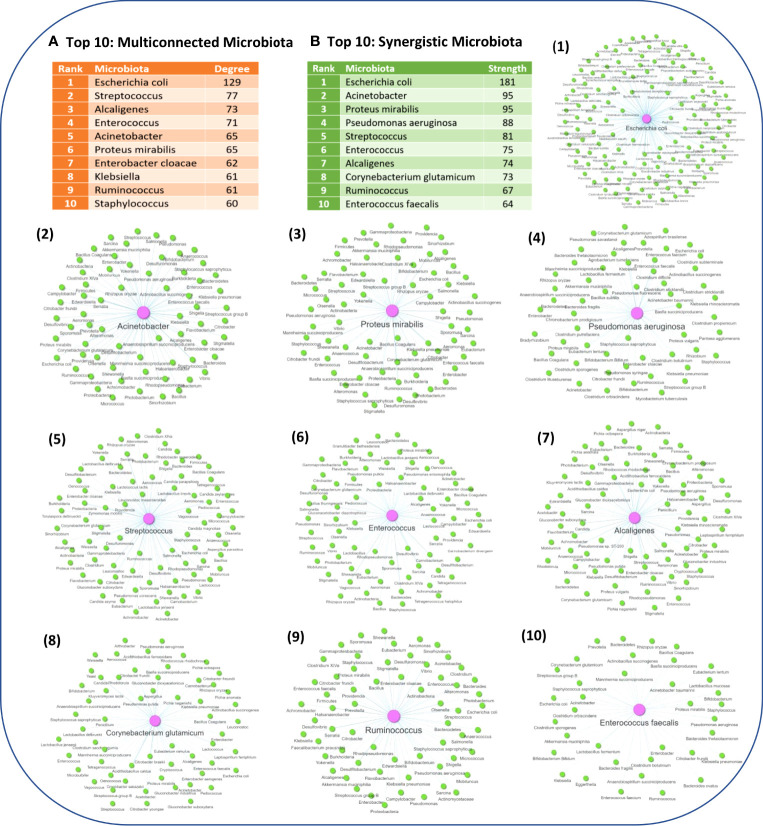
**(A)** The Top 10 microbiota components with the highest degree of pairwise similarity of metabolites that are also produced by another microbiota component. **(B)** The Top 10 microbiota components with the highest strength topology, thus having the highest potential to exert synergistic effects. Figure also illustrates the associated microbiota pairs of each of the Top 10 synergistic microbiota components as a microbiota-to-microbiota association network.

Network analysis also allowed to identify the microbiota components which have the highest degree of pairwise similarity of metabolites which are also produced by other microbiota components, with the Top 10 high degree indicated in **Figure 5A**. Interestingly, almost all of the Top 10 high degree microbiota components are either known human pathogenic or opportunistic pathogens, with the exception of *Ruminococcus* genus which are not considered to be opportunistic pathogens. However, several members of the *Ruminococcus* genus are commensal gut microflora and alternations in their abundance has been found in several NDs, including MS (72, 73), PD (74–76), AD (77) and ALS (78), but also other diseases such as Chron’s disease (79–81). *Escherichia coli* genus which has the highest degree of pairwise similarity of metabolites which are also produced by other 129 microbiota is also a commensal gut microbiota, but it can become pathogenic when it infects other tissues. Moreover, several of the other high degree microbiota components are also commensal microbiota that can become pathogenic when their abundance increases or they migrate to other tissues, such as the genera *Staphylococcus*, *Streptococcus*, *Alcaligenes* and *Enterococcus*. Therefore, the network analysis results indicate that human pathogenic bacteria have a high degree of pairwise similarity with other microbiota components, which not only allows them to influence multiple ISPs, but also allows them to exert synergistic immunomodulatory effects. In addition, **Figure 5B**, indicates the Top 10 microbiota components which have the highest node strength, which is the sum of weights of the links connected to the node, hence node strength indicates the microbiota components with the highest potential of synergistic immunomodulatory effects.

### 3.5 Possible microbiome-host-immune system interactions in NDs

Perturbations in microbiome-immune system interactions have been associated with the development of NDs (82, 83). Therefore, to investigate the possible role of the immunomodulatory effects of microbiota-metabolic products in NDs we first identified for each of the four NDs (AD, PD, MS and ALS) the number of disease-associated genes that can be also targeted by microbiota components *via* their metabolic products, which are termed intersections nodes. More specifically, we identified 68, 91, 72 and 93 intersection nodes in MS, AD, ALS and PD, respectively. **Table 4**, indicates the number of genes out of the top 200-disease associated genes included in the analysis for each ND, which can act as intersection nodes, including also the number of microbiota components and their metabolic products that interact with these intersection nodes. The results indicate that AD and PD have the highest number of disease-associated genes that are targeted by microbiota-derived metabolites, as they have 91 and 93 intersection nodes, respectively. In addition, the number of microbiota components that can interact with these intersection nodes is also higher in AD and PD compared to MS.

**Table 4:** Characteristics of the interaction of microbiota and their metabolites with NDs-associated genes.

	PD	MS	ALS	AD
Microbiota nodes that produce metabolites that interact with the intersection nodes	157	107	141	158
Metabolites nodes that target the intersection gene nodes	57	33	50	54
Intersection gene nodes	93	68	72	61

Moreover, through the reconstruction and visualization of the MMDI networks for the four NDs, we were able to identify specific disease-associated genes associated with ISPs that are also interactors and modulated by microbiota *via* metabolite-to-gene interactions. **Table 5** indicates for each of the four MMDI networks the number of intersection nodes that are associated with ISPs and the number of microbiota nodes and their metabolic products that interact with the ISP- associated intersection nodes. These results indicate that although MS has the least number of disease-associated genes that are targeted by microbiota-derived metabolites, the majority of these genes are associated with GO ISP enriched terms. On the contrary, in AD and PD which have the highest number of disease-associated genes that are targeted by microbiota-derived metabolites, only approximately one-third of these intersection nodes are also associated with GO ISPs. In addition, in ALS less than one-third of intersection genes are associated with GO ISP enriched terms. However, the results also indicated that these GO ISP- associated intersection nodes in each of the four NDs can influence either all or the majority of the GO ISP terms associated with these diseases. More specifically, the GO ISP-associated intersection nodes participate in 295 out of the 303, 63 out of the 63, 28 out of the 30 and 95 out of the 97 ISPs, in MS, PD, ALS and AD, respectively. This suggest that the GO ISPs-associated intersection nodes which are targeted by microbiota have pleiotropy in ISP effects, which possible allows microbiota to affect multiple disease related ISPs.

**Table 5:** Characteristics of the immunomodulatory effects of microbiota and their metabolites in the MMDI networks.

	PD	MS	ALS	AD
Microbiota with immunomodulatory effects	108	94	84	104
Microbiota-derived metabolites that interact with GO ISPs	30	27	26	29
Intersection nodes that are associated with GO ISPs	30	50	22	32
GO ISPs associated with each disease	63	295	28	95

**Figure 6:**
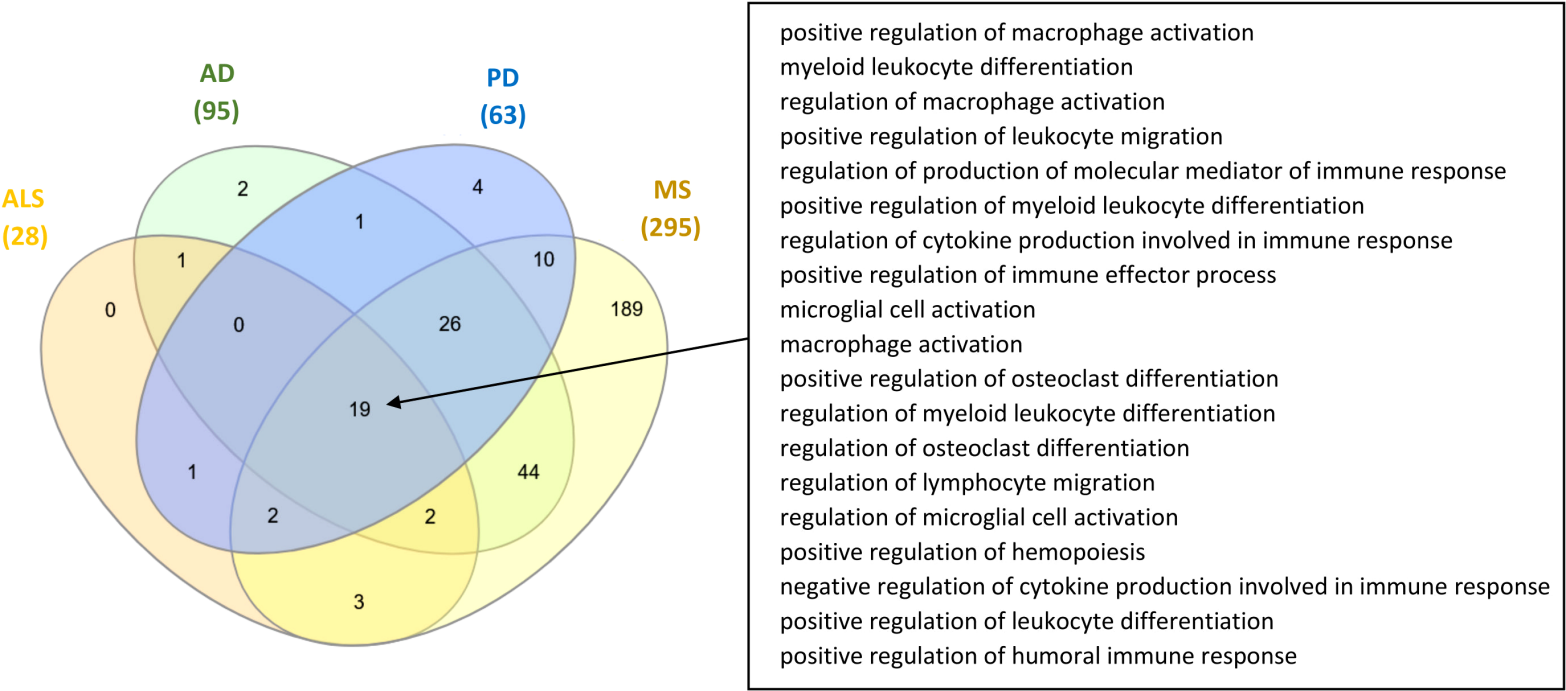
Comparison of the GO ISPs that can be modulated by microbiota components in the four ND states AD, MS, ALS and PD, highlighting the 19 common GO ISPs.

Comparison between the microbiota components and microbiota-derived metabolites that have been identified to influence the ISP-associated intersection nodes for the four NDs, indicated that there are 65 common microbiotas and 21 common metabolites between all four NDs. In addition, comparison between the GO ISPs that can be modulated by microbiota in the four ND states indicated that they share 19 common GO ISPs, that can be modulated by microbiota components, shown in **Figure 6**.

### 3.6 Network re-wiring of the immunomodulatory effects of microbiota in NDs

The immune system at equilibrium has a certain set of interactions and nodes, whereas at disequilibrium new nodes and interactions will emerge due to the activation of ISPs associated with the inflammatory trigger or the disease state. This dynamic change of immune system molecular interactions gives rise to specific disease immune phenotypic profiles. This “re-wiring” in the network of molecular interactions due the presence of a disease state could be also reflected in the re-wiring in the networks of microbiota-host interactions. Therefore, it is expected that the effect of the same microbiotas might be different in different diseases due to the different immune profiles and different perturbations that will emerge from microbiota-host interactions. This is because immune system re-wiring due to a disease condition will lead to the emergence of new interactions and nodes which would also change the immunomodulatory effects of microbiota-host interactions.

**Figure 7:**
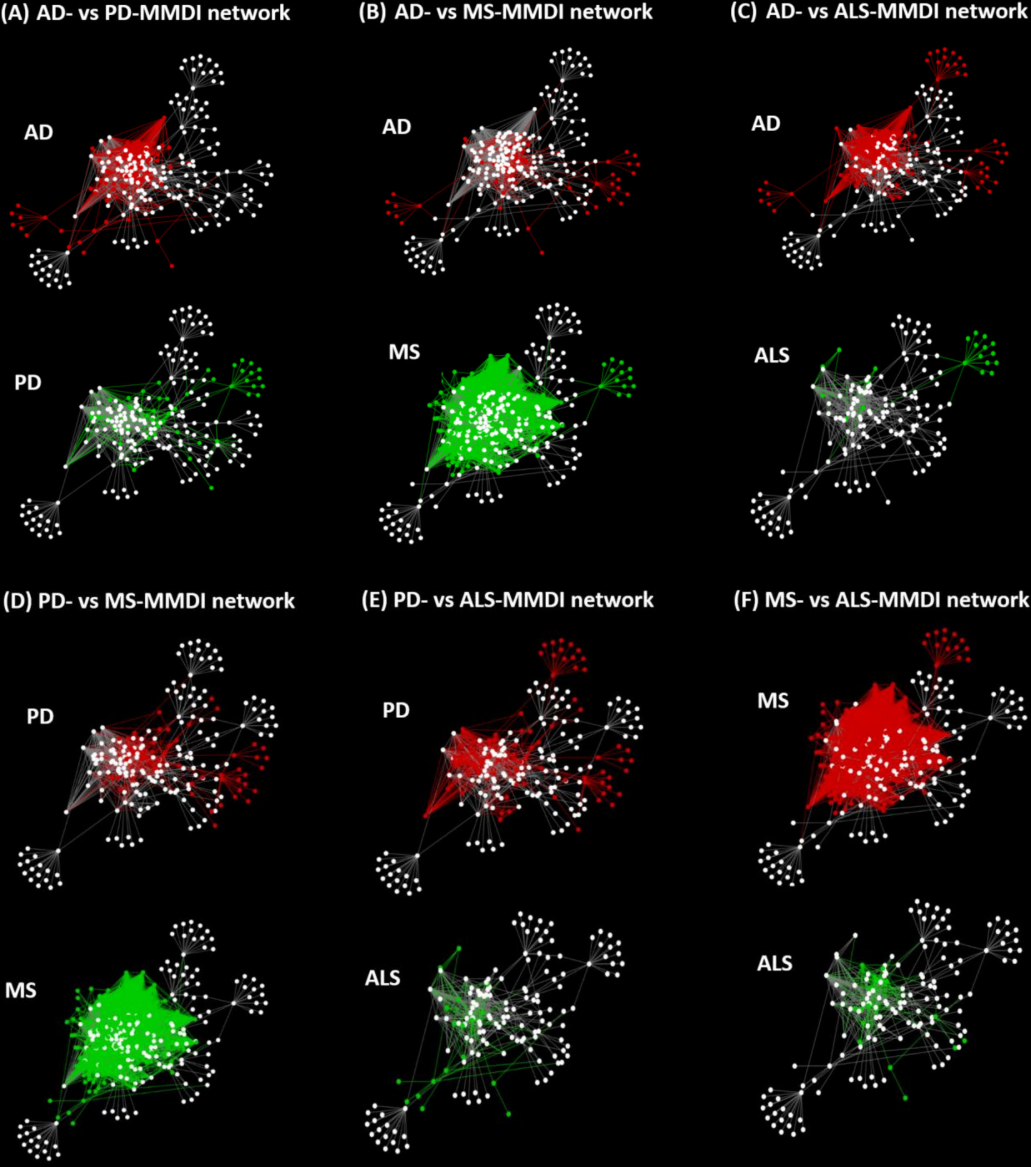
Pairwise network comparisons between the MMDI networks, with red nodes/edges indicating nodes only present in one network and green nodes/edges indicating nodes that are only present in the other network, whereas white nodes indicate common nodes between the two networks.

To investigate the level of re-wiring of the immunomodulatory effects of microbiota *via* metabolite-gene interactions under the four NDs we used the *DyNet* app in Cytoscape, where we performed pairwise network comparison which allowed to identify nodes and edges that differ between two networks. **Figure 7**, illustrates the pairwise comparison between all the pairs of MMDI networks. The pairwise comparison between the four MMDI networks, indicated that MS compared to the other three NDs (ALS, PD and AD) has a higher level of network re-wiring due to the presence of several nodes and edges that are not present in the other three networks. In addition, the pairwise comparison indicated that ALS has the least level of network re-wiring compared to the other three NDs (AD, PD and MS). Moreover, the comparison indicated that the level of re-wiring between AD and PD is not high, as they share several common nodes and edges, and have relatively few different nodes and edges.

### 3.7 Possible contribution of microbiota - EBV interactions in the development of NDs

Both microbiota and viruses have the ability to influence ISPs, with microbiota exerting their immunomodulatory effects *via* their metabolic products and viruses through their viral proteins. In addition, both viruses and microbiota have been associated with the development of NDs, hence microbiota-virus interactions might contribute to the development of NDs as well. Viruses, through PPIs can manipulate host ISPs and lead to their dysregulation, which might contribute to the development or progression of NDs. Therefore, we explored whether microbiota components that have immunomodulatory effects can modulate similar ISPs as viruses associated with NDs. To explore these similarities, we used the case of EBV which is associated with the development of three NDs (AD, PD and MS) and because EBV is a well-studied virus that has lots of available PPI data.

**Figure 8:**
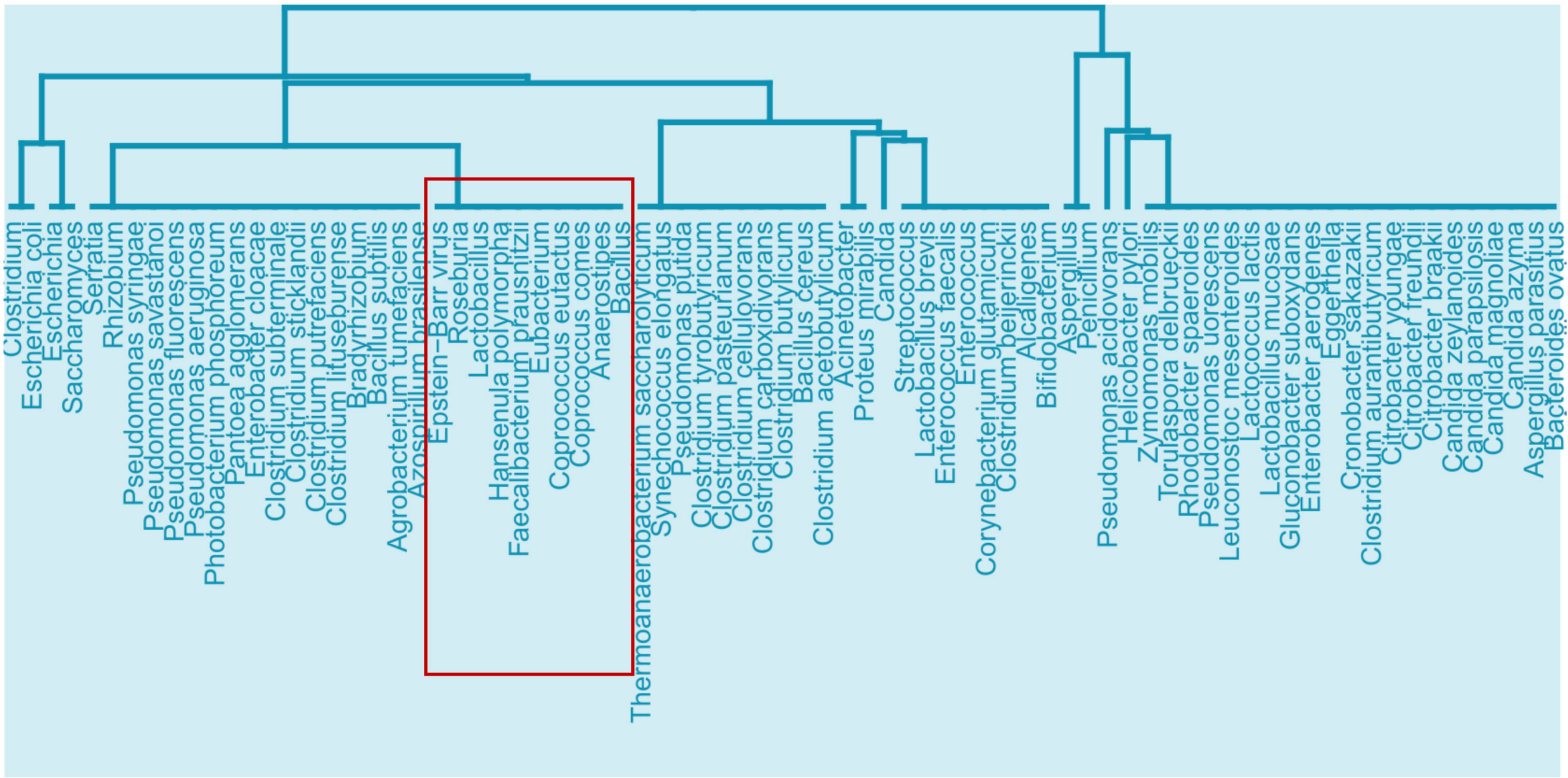
Cluster from the similarity results of EBV with the 241 microbiota that can affect the 24 ISPs that can be modulated by both microbiota components and EBV, highlighting the branch that EBV belongs to and the microbiota components that can also affect all 24 of the common GO ISPs.

We first performed enrichment analysis on the 1247 human protein targets of EBV proteins to investigate the effects of EBV in general, which revealed 53 significantly enriched GO ISP terms. Then we compared the EBV significantly enriched GO ISP terms with the 120 GO ISP terms associated with microbiota from the MMI network, which revealed 24 common GO ISPs that can be modulated by both EBV and the 241 microbiotas. Subsequently by using the microbiota-to-GO ISPs projection of the MMI network we extracted and identified the microbiota components which can influence these 24 GO ISPs which are also modulated by EBV. Then we performed similarity-based analysis between the 241 microbiotas and EBV to group them into clusters based on their ability to modulate these 24 common GO ISPs. The clustering results indicated the presence of 24 clusters of microbiotas. The clustering results (see **Figure 2** of **Supplementary File 1**) indicated that 9 out of the 241 microbiotas belong to the same cluster with EBV and thus, like EBV, they can modulate all of the 24 GO ISPs, **Figure 8**. More specifically these microbiota components are: *Roseburia* genus, *Lactobacillus* genus, *Hansenula polymorpha* species, *Faecalibacterium prausnitzii* species, *Eubacterium* genus, *Coprococcus comes* species, *Coprococcus eutactus* species, *Anaerostipes* genus and *Baccillus* genus. *Lactobacillus* are considered as “friendly” bacteria that live symbiotically within the human host exchibiting health promoting effects and protect against pathogenic organisms (84). In addition, the *Lactobacillus* and *Bacillus* genera are found in probiotic supplements, with *Lactobacillus* being the most common genus used for probiotics (85, 86).

However, as already mentioned, pathogen-host interactions in disease states differ from healthy state due to the emergence of new interactions and processes that arise based on the pathological mechanisms activated as a result of the disease. Therefore, in order to investigate the possible microbiota-EBV interactions in modulating GO ISPs associated with NDs, we first created three integrated EBV-ND PPI networks, where the EBV-host PPI network was merged with the disease-associated proteins of each of the three NDs (AD, PD and MS). Then, to identify GO ISPs that can be modulated by EBV in each of the three NDs, we performed enrichment analysis on the human protein targets of EBV proteins and their first neighbors in each of the integrated EBV-ND PPI networks. The enrichment analysis of EBV PPI in NDs, indicated that the number of significantly enriched GO ISPs that can be modulated by EBV in MS disease states are 235, in PD state are 87 and in AD state are 99.

Then we compared the EBV GO ISP enriched results in the three NDs, with the GO ISPs that can be modulated by microbiota components in the three ND states, which allowed to identify for each ND state the GO ISPs that can be modulated by both microbiota components and EBV. The comparison between the immunomodulatory effects of EBV and microbiota in MS disease, indicated that 169 MS-associated GO ISPs can be modulated by both microbiota components and EBV. Similarly, comparison of the immunomodulatory effects of EBV in PD and AD diseases with the immunomodulatory effects of microbiota on the GO ISPs associated with these disease states, indicated 18 and 36 common ISPs that can be modulated by both microbiota components and EBV, respectively. These results indicate that in MS there is a higher number of disease-associated ISPs that can be modulated by both microbiota and EBV infection, than in PD and AD. Therefore, it is possible that microbiota-EBV interactions, in terms of their immunomodulatory effects, might play a more significant role in MS pathogenesis rather than PD and AD.

## 4 Discussion

In this study we performed a network-based bioinformatics approach with the aim to first investigate the immunomodulatory effects of microbiota (bacteria and fungi) through their metabolic products. We also explored how various microbiota components, through their immunomodulatory effects, might be able to influence ISPs associated with NDs, and thus possibly contribute to their development and/or progression. In addition, we explored the immunomodulatory effects of EBV, which is associated with the development of the three NDs, MS, AD and PD, both in general and ND specific states. Moreover, we examine how microbiota-virus interactions might contribute to the modulation of ISPs associated with pathogenic mechanisms involved in NDs. Our network-based methodology is summarized in **Figure 1**.

Statistically significant enrichment analysis revealed 120 GO ISP terms that can be modulated by 93 microbiota-derived metabolites through their interaction with 542 human gene targets. The GO ISPs results, **Table 3**, indicated that 20.49% of the GO ISP terms belong to the group of positive regulation of leukocyte migration. During tissue damage or infection migration of circulating blood leukocyte cells are an important component in the elimination of the inflammatory triggers and tissue repair process (87). Leukocyte migration is also essential for the resolution of inflammatory processes and uncontrolled migration of leukocytes cells is observed in inflammation and NDs. Therefore, microbiotas through their metabolic products can influence immune responses during inflammatory triggers.

In addition, 7.38% of the enriched terms belong to the group of myeloid leukocyte differentiation. Myeloid cells are important in mounting effective inflammatory responses during viral infections (88). Therefore, microbiota by affecting myelopoiesis can possibly affect innate immune responses and the formation of immunological memory against viral infections.

Moreover, 14.75% of the GO ISP terms belong to the group of complement activation, classical pathway, which plays an important role in innate immune system defenses against pathogens and also complements antibody responses against pathogens by the adaptive immune system. The complement system also plays a critical role in commensal microbiota-immune system symbiosis and health homeostasis. Improper complement system recognition of commensal microbiota as pathogenic would lead to excessive immune responses and hence to the emergence of immune-mediated diseases (60, 61). Therefore, microbiota, by interfering with the complement system, not only have the ability to affect both innate and adaptive immune responses against pathogens, but also regulate host responses against symbionts.

The enrichment analysis results also indicated that microbiota can affect the formation of memory B and T cells by modulating the signaling pathways that are involved in the differentiation of B and T cells, as 11.48% of the GO ISPs terms belong to the group of antigen receptor-mediated signaling pathway. In addition, the enrichment results indicated that microbiota can affect humoral immune response mediated by circulating immunoglobulin. This suggests that microbiota can affect antibody production. This is supported by evidence that indicates that gut commensal microbiota can affect the production of IgA, and the production of autoantibodies (64, 65). Evidence also indicates that SFCAs, which are produced by gut microbiota from dietary intake, can influence antibody production. Reduced intake of dietary fiber leads to the production of low levels of SFCAs, and this was shown to result in defective pathogen-specific antibody responses (89). Autoantibodies are a common feature of autoimmune diseases, but also of NDs, like MS, therefore improper diet in combination with altered microbiota composition might lead to the loss of self-tolerance and impaired immune responses to pathogenic organisms.

Furthermore, 1.64% of the terms belong to the group of microglia cell activation. Gut microbiota can affect the maturation and activation of microglia cells *via* the production of SCFAs (67) and microbiota-derived NTs (68). These microbiota-mediated effects on microglia were shown to also affect innate immune responses in the CNS against viral infections, mediated by the production of SCFAs (67). Microglia dysfunction has been implicated in the pathogenesis of several NDs, including MS, AD, PD and ALS, as they contribute to neuroinflammation (90). Altered microbiota composition has also been associated with the development of NDs, therefore microbiome-microglia interactions might influence NDs pathogenesis (68). Viruses which are also associated with the development of NDs and can also interfere with microglia functions (91). Therefore, since alternation in microbiota can influence microglia immune responses against viral infection, microbiota-virus-microglia interactions could also influence the pathogenesis of NDs.

The next step of our methodology involved the reconstruction and visualization of the MMI network that contained microbiota - metabolites - GO ISP terms interactions. From the MMI network, we extracted three network projections that involve the direct relationships between (a) metabolites and GO ISPs, (b) microbiota and GO ISPs and (c) microbiota and metabolites, illustrated in **Figure 2**. Projections (a) and (b) were used to identify clusters of microbiota-derived metabolites and clusters of microbiotas genera/species/strains that have similar or dissimilar immunomodulatory effects, based on the GO ISPs that they can affect. The similarity analysis results of the microbiota-derived products indicated that the majority of the microbiota-derived NTs belong to the same cluster, therefore they exert similar immunomodulatory effects. NTs can influence both innate and adaptive immune responses, with leukocyte cells expressing receptors for several NTs including glutamate, dopamine, serotonin and acetylcholine (92, 93). A bidirectional cross-talk exists between the brain and the peripheral immune system as leukocyte can also synthesize and released NTs and they can also produce cytokines that participate in the neuroimmunomodulatory circuitry (93). Therefore, NTs produced by microbiota can indirectly modulate neuroinflammation and they can also possibly affect neural regulation of innate immunity, including microglia activation (68). Interestingly, evidence indicates that microglia have also the capacity to be ‘primed’ based on their history of inflammatory stimuli and develop innate immune memory (94). Depending on the initial stimuli, repeated exposure to secondary inflammatory stimuli may enhance their responses or lead to loss of responsiveness (94). This suggests that a form of classical conditioning learning might exist in the pairing of microglia with the initial stimuli that influences responses during re-exposure. In a mouse model of AD, it was shown that application of a peripheral stimuli caused innate immune memory training of microglia cells in the brain which exacerbated β amyloidosis, whereas innate immune tolerance alleviated this effect (95). Therefore, it is possible that microbiome-derived NTs and SFCAs, which can modulate microglia maturation and activation, might also affect the priming of microglia towards inflammatory stimuli and thus the development of innate immune memory by microglia, which might contribute to NDs development.

In addition, the clustering results also indicated that groups of microbiota can influnce the same ISPs. However it does not necessarily mean that by targeting the same ISP the outcome of their effect would be the same as they might interact with different genes in these ISPs, thus leading to either inhibition or activation of the ISPs. Therefore, in order to identify microbiota that have the same outcome on an ISP and thus possible synergistic effets, we extracted from the MMI network the direct relationships between microbiota and metabolites, **Figure 2C**. Then by using the microbiota to metabolites relationships we created a microbiota-to-microbiota associations network based on pairwise similiarity to identify pairs of microbiota components that can produce the same metabolites. Topological analysis of the microbiota-to-microbiota associations network indicated the Top 10 microbiota components that had the highest degree of pairwise similarity with metabolites produced by other microbiota components. Almost all of the Top 10 microbiota components were human pathogenic or opportunistic pathogens, with the exception of *Ruminococcus* genus. This suggests that pathogenic bacteria by producing several metabolites that can be also produced by other microbiota components are able to influence multiple ISPs effects. This also allows them the potential to exert synergistic immunomodulatory effects with other microbiota components, which possibly provides them with increased survival and pathogenicity. The potential of synergistic actions between microbiota components through their metabolic products can possibly affect the immune responses towards the emergence of a specific immune system phenotype. This can occur when there is either an increase or a decrease in the abundance of microbiota components that produce the same metabolite. However, the outcome of the immunomodulatory effects influenced by commensal microbiota composition is not only determined by the combinatorial action of their synergistic relationships, but also by their antagonistic relationships. The metabolites produced by one microbiota component can have an opposite effect on the host’s ISPs compared to the effect of the metabolic products produced by another microbiota component, thus leading to the emergence of antagonist relationships.

The reconstruction and visualization of microbiota-host-interactions in NDs (AD, ALS, PD and MS), allowed to identify mechanisms by which microbiota components *via* their metabolic products might influence pathologies associated with NDs, **Table 4**. More specifically, it allowed to identify intersection nodes, which are nodes that are ND-associated genes but they are also genes that are targeted by microbiota components *via* their metabolic products. In addition, it allowed to identify intersection nodes which are associated with GO ISPs in these four ND states, **Table 5**. The results indicated that although MS has the least number of disease-associated genes that are targeted by microbiota-derived metabolites, the majority of these genes are associated with almost all of the GO ISPs associated with MS disease. On the contrary, in AD and PD where microbiota had the highest number of disease-associated genes that are targeted by microbiota-derived metabolites, only approximately one-third of these intersection nodes are also associated with disease-associated GO ISPs. This possibly suggests that the influence of microbiota on GO ISPs-associated with MS disease is higher than in AD and PD, thus microbiota might have a more significant role in MS disease inflammation. However, this does not mean that microbiota do not play a role in the pathogenesis of AD and PD. At least, the impact of microbiota in these diseases seems not to be mediated by their immunomodulatory effects but it can be possibly mediated by the dysregulation of other host processes. Moreover, comparison between the microbiota components and microbiota-derived metabolites that influence ND- associated ISPs in the four ND states indicated that there are 65 common microbiotas and 21 common metabolites between all four NDs, as well as 19 common GO ISPs, indicated in **Figure 6**.

However, although these four NDs share common microbiota and metabolites it does not mean that the immunomodulatory effects of these microbiotas are the same in these diseases as network dynamics change, due to the emergence of new interactions and nodes that stem from the disease pathology. This “re-wiring” between molecular interactions due to the presence of a disease state is also expected to change microbiota-host interactions. We investigated network re-wiring of the immunomodulatory effects of microbiota between the four NDs (AD, ALS, PD and MS) by performing pairwise network comparison which allowed to identify nodes and edges that differ between two network pairs of NDs, **Figure 7**. The pairwise comparison between the four MMDI networks, indicated that MS compared to the other three NDs (ALS, PD and AD) has a higher level of network re-wiring due to the presence of several nodes that are not present in the other three networks. In addition, the network re-wiring analysis indicated that ALS has the least level of network re-wiring compared to the other three NDs (AD, PD and MS). Moreover, the comparison indicated that the level of difference between AD and PD is not high, as they share several common nodes and edges having only relatively few different nodes and edges.

Finally, to investigate the possible contribution of microbiota-virus interactions in the development of NDs, we explored whether microbiota that have immunomodulatory effects can modulate similar ISPs as EBV which is associated with the development of three NDs: AD, PD and MS. To investigate the possible presence of microbiota-EBV interactions in modulating GO ISPs associated with NDs, we first constructed three integrated EBV-ND PPI networks, where the EBV-host PPI network was enriched with the disease-associated proteins of each of the three NDs (MS, PD and AD). The enrichment analysis of EBV PPI interactions in NDs, indicated 235, 87 and 99 significantly enriched GO ISPs that can be modulated by EBV in MS, PD and AD states, respectively. Comparison of the EBV GO ISPs results in the three NDs, with the ISPs that can be modulated by microbiota components in the three NDs states, allowed to identify for each ND the ISPs that can be modulated by both microbiota components and EBV. The comparison results indicated that 160 GO ISPs associated with MS disease can be modulated by both microbiota components and EBV, whereas only 18 and 36 GO ISP can be modulated in PD and AD, respectively. This possibly indicates that microbiota-virus immunomodulatory-related interactions, might play a more significant role in MS disease pathogenesis rather than in the pathogenesis of PD and AD.

## 5 Conclusion

In this paper, we provided a bioinformatics insight approach that tries to capture the effects of microbiota, bacteria and fungi, in shaping immune responses and influencing the formation of immunological memory cells through their metabolic products, under ND states and under ND states with EBV viral infection. We recognize that our study might have certain limitations mainly based on the completeness and the possible biases of the databases used, however despite these limitations our approach allowed us to formulate the following conclusions.

The enrichment analysis of microbiota-host interactions allowed to highlight various aspects of the innate and adaptive immune response systems that can be modulated by microbiota, which includes responses during inflammatory triggers. The results also indicated that microbiota can influence the activation and maturation of microglia which are implicated in the development of NDs.

The reconstruction of the microbiota-to-microbiota associations network based on pairwise similiarity of pairs of microbiota components that can produce the same metabolites allowed to possibly identify a potential of synergestic immunomodulatory actions between microbiota components. The pairwise similarity also indicated that known pathogenic bacteria, such as the *Escherichia coli* and *Klebsiella* genera, that can produce several metabolites that are also produced by other microbiota components, allowing them to influence multiple ISPs and thus possibly contributing to their pathogenicity.

Investigation of possible microbiota-host-immune system interactions in NDs allowed for the isolation of specific microbiota components and metabolic products that interact with disease-associated genes that participate in ISPs. The results also suggest that the impact of microbiota-derived metabolites in influencing ISPs is higher in MS, than AD, PD and ALS.

Finally, investigation of the possible contribution of microbiota- virus interactions in the development of NDs, allowed to identify ISPs that can be modulated by both microbiota components and EBV. The result of the analysis also suggests that the combinatorial action of the immunomodulatory effects of microbiota-EBV interactions might play a more significant role in MS disease pathogenesis rather than in the pathogenesis of PD and AD.

## Data availability statement

Publicly available datasets were analyzed in this study. This data can be found here: HMDB https://hmdb.ca/downloads, PHISTO https://phisto.org/, VirHostNet https://virhostnet.prabi.fr/, DISEASES https://diseases.jensenlab.org/Search.The in-house developed HMDB parsing R script can be provided upon request to the authors.

## Author contributions

AO and GS have contributed to the conceptualization, methodology, review and editing of the manuscript. AO collected and analyzed the data, and wrote the original draft manuscript, under the supervision of GS. All authors have contributed to the article and have read and agreed to the published version of the article.

## Funding

This study received support from the Cyprus Institute of Neurology & Genetics and funded by Telethon.

## Conflict of interest

The authors declare that the research was conducted in the absence of any commercial or financial relationships that could be construed as a potential conflict of interest.

## Publisher’s note

All claims expressed in this article are solely those of the authors and do not necessarily represent those of their affiliated organizations, or those of the publisher, the editors and the reviewers. Any product that may be evaluated in this article, or claim that may be made by its manufacturer, is not guaranteed or endorsed by the publisher.
