# Supplementary material for: Immunomodulatory effects of microbiota-derived metabolites at the crossroad of neurodegenerative diseases and viral infection: network-based bioinformatics insights
Source: Front Immunol. 2022 Jul 19;13:843128. doi: 10.3389/fimmu.2022.843128 (PMC9344014; doi:10.3389/fimmu.2022.843128)
Supplement: Supplementary file 1 [file DataSheet_1.docx]

**
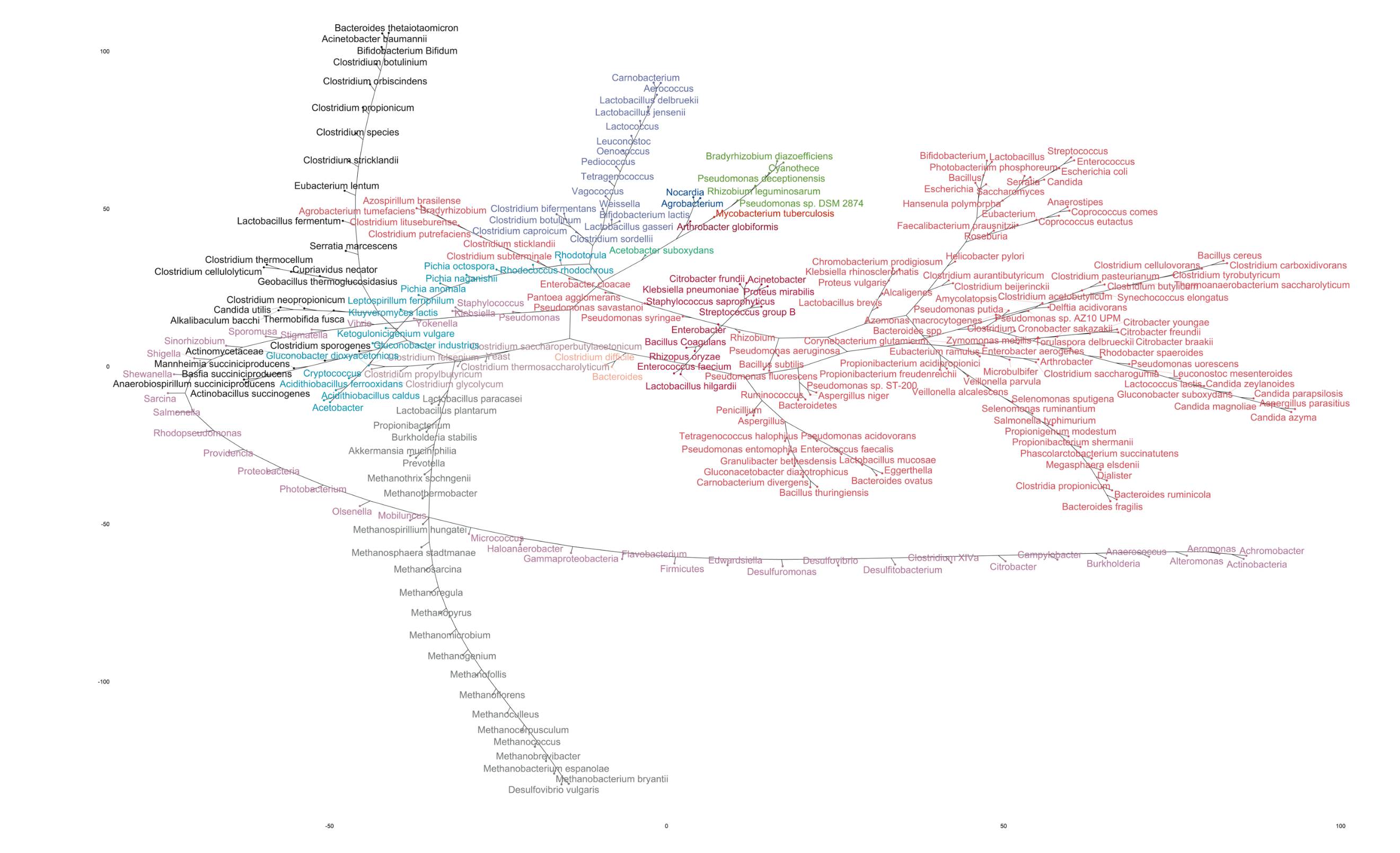
Supplementary Figure 1:** Phylogenetic layout of the agglomerative hierarchical clustering results of the 256 microbiotas based on GO ISPs terms similarity

**Supplementary File 1**


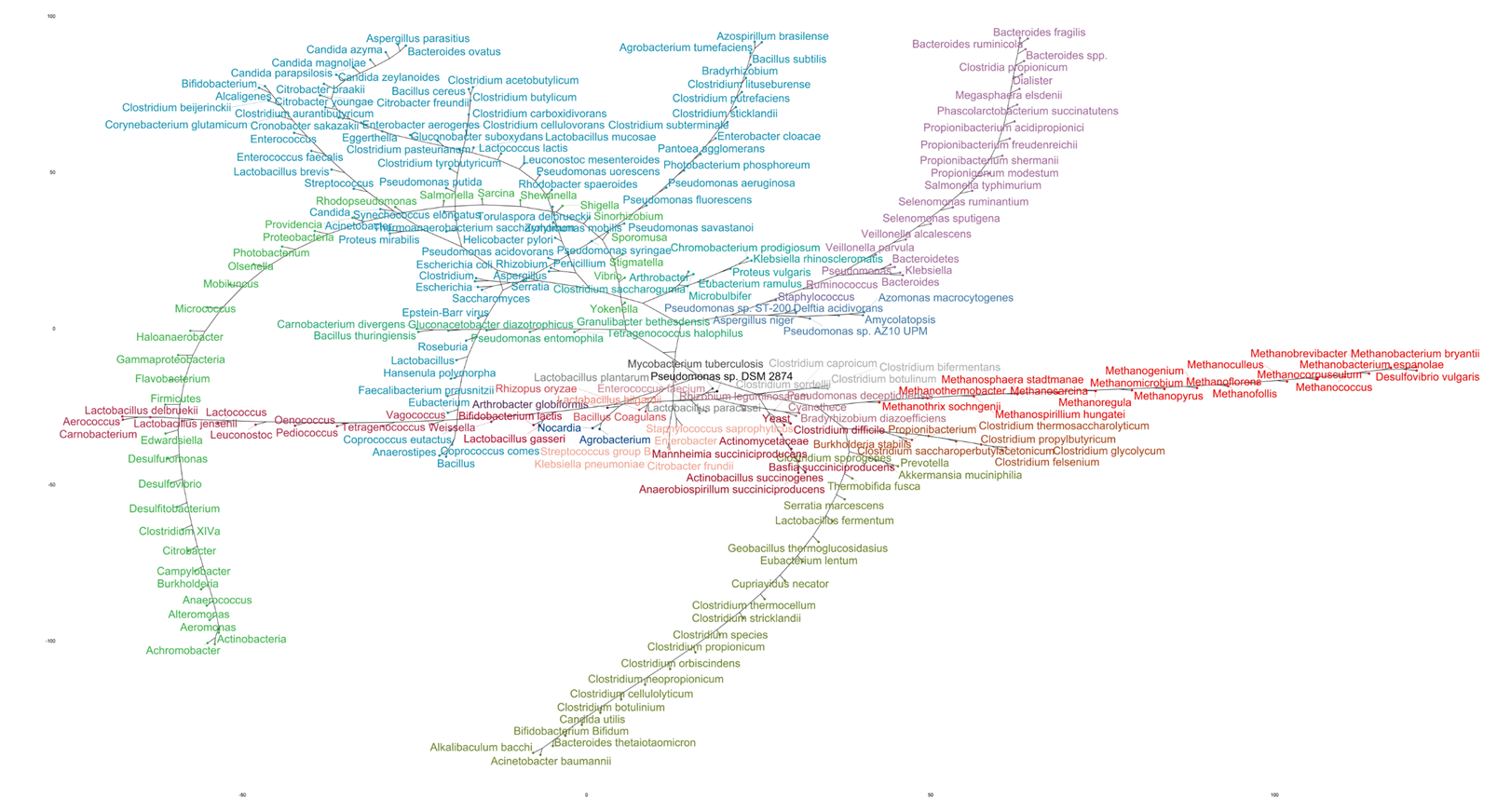
**Supplementary Figure 2:** Phylogenetic layout of the agglomerative hierarchical clustering results of the 241 microbiotas that can influence the same GO ISPs as EBV in general.
